# A Generalized Approach to the Modeling and Analysis of 3D Surface Morphology in Organisms

**DOI:** 10.1371/journal.pone.0077551

**Published:** 2013-10-25

**Authors:** Janice L. Pappas, Daniel J. Miller

**Affiliations:** Museum of Paleontology, University of Michigan, Ann Arbor, Michigan, United States of America; Monash University, Australia

## Abstract

The surface geometry of an organism represents the boundary of its three-dimensional (3D) form and can be used as a proxy for the phenotype. A mathematical approach is presented that describes surface morphology using parametric 3D equations with variables expressed as *x*, *y*, *z* in terms of parameters *u*, *v*. Partial differentiation of variables with respect to parameters yields elements of the Jacobian representing tangent lines and planes of every point on the surface. Jacobian elements provide a compact size-free summary of the entire surface, and can be used as variables in principal components analysis to produce a morphospace. Mollusk and echinoid models are generated to demonstrate that whole organisms can be represented in a common morphospace, regardless of differences in size, geometry, and taxonomic affinity. Models can be used to simulate theoretical forms, novel morphologies, and patterns of phenotypic variation, and can also be empirically-based by designing them with reference to actual forms using reverse engineering principles. Although this study uses the Jacobian to summarize models, they can also be analyzed with 3D methods such as eigensurface, spherical harmonics, wavelet analysis, and geometric morphometrics. This general approach should prove useful for exploring broad questions regarding morphological evolution and variation.

## Introduction

The world is filled with organisms that display a broad diversity of surface morphologies. From the rippled texture of a tropical mollusk shell to the exquisitely delicate frustule of a diatom, surfaces are everywhere. It is surprising that so few studies have sought to rigorously describe the surface geometry of biological forms. The fact that the surface of an organism represents the boundaries of its three-dimensional (3D) morphology means that surface geometry can be used as a proxy for the phenotype. Studying surfaces may lead to a deeper understanding of the function and evolution of biological structures. As organisms evolve, so too may their surfaces, and studying the spatiotemporal distribution of surface morphology may lead to a greater understanding of evolutionary processes. Surfaces often change during growth, and their study may provide a different way of describing patterns of ontogeny.

While a great deal of effort has been devoted to the quantitative measurement of organisms, most studies have resorted to the measurement of distances between points or outlines. Often measurements are made from two-dimensional (2D) projections of 3D objects. Advances in 3D scanning and imaging technologies have generated great interest in the digitization of biological form, and 3D data have been incorporated in studies using methods such as eigensurface analysis [1], [2], spherical harmonics [3], wavelet analysis [4], and geometric morphometrics [5]. Some studies in pattern recognition and computer visualization have described methods that emphasize using point clouds in shape matching of 3D surfaces [6], [7]; other studies are concerned with automating shape matching in 3D space [8].

We present a new approach to characterize 3D surfaces that differs in an important respect from existing methods. We begin with a parameterized 3D surface model rather than an empirically-derived array of landmarks or a point cloud from an actual specimen. Consequently, our approach permits the generation of theoretical or abstracted morphologies and this, in itself, presents some interesting opportunities for the study of biological form. However, while model-based, our method is fully capable of yielding empirical results, and we demonstrate how morphologically accurate models of real forms can be produced using reverse engineering principles [9]. That is, we can create a model from the empirical object, but numerical representation is extracted from equations of the model rather than directly from the empirical object itself. From our models, we calculate a matrix of values summarizing the entire surface, the elements of which can be used in conventional multivariate ordinations to generate surface morphospaces.

Our broader objective is to develop a generalized approach that can be used to study a wide variety of morphologies and morphological evolution in a more mathematically rigorous and flexible way. Our approach can be used alone or in conjunction with other analytical methods for the study of forms, both theoretical and empirical, and be applied to problems ranging from large scale macroevolution to small scale intrapopulation variation. Using examples from the Mollusca and Echinodermata, we show how forms with different morphologies and taxonomic affinities can be plotted meaningfully in a common morphospace. We also provide an example of how to study ontogenetic trajectories, real or theoretical, including allometric and isometric growth.

### 1.1. Mollusks and Echinoderms as Test Organisms

Both mollusks and echinoderms are taxonomically rich, well-studied, and have a well-documented fossil record. These phyla are suitable for studies of ecological and evolutionary patterns and processes over long time spans. The external morphology of many shelled mollusks has been the focus of numerous ecological and evolutionary studies, and the distinct spiral geometry of many forms has long attracted the interest of both mathematicians and biologists [10]–[11], providing an ideal test case for our approach. Others have analyzed echinoid form mathematically [12]–[13] and we show how our approach can be used on this group as well.

The study of spiral form in mollusks was codified by Thompson [14] early in the 20^th^ century. However, the prominent spiral geometry of many mollusks was studied even earlier by Moseley [15]–[16], and his studies were the basis for Raup's [17]–[20] classic analyses of mollusk shells using a generating curve that moves along a logarithmic spiral with a fixed reference frame. Raup's model stimulated a number of additional studies [21]–[23], and several modifications were introduced, including the use of a moving reference frame [24]–[26], multiple helico-spirals [27]–[28], and the explicit inclusion of growth and morphogenesis [29]–[32].

For models with a generating curve, the shell surface is an epiphenomenon that reflects the cumulative history of the position of the generating curve as it moves along the spiral. These models, although treating the surface as a byproduct of growth, nevertheless succeed in simulating the broadest aspects of marginal accretion. However, describing mathematical properties such as the generating curve, type of spiral, and reference frame required to produce a coiled surface is not the same as describing the surface as an entity. Shell growth develops via marginal accretion, but the resulting form is often what interacts with the environment and can be the target of selective pressure. Thus, although we might be able to reduce a shell mathematically to marginally accreting spiral growth, morphological evolution involves the whole shell, and there is a need for methods that describe the surface phenotype as completely and directly as possible.

The objective of theoretical coiling models, in part, is to produce a low dimension morphospace with axes that represent particular parameters of the model. In contrast, our approach is to generate 3D theoretical models of whole surfaces that are then analyzed in an empirical *n*-dimensional space where the meaning of the axes is determined *a posteriori*.

### 1.2. Background on 3D surface geometry

**Figure 1:**
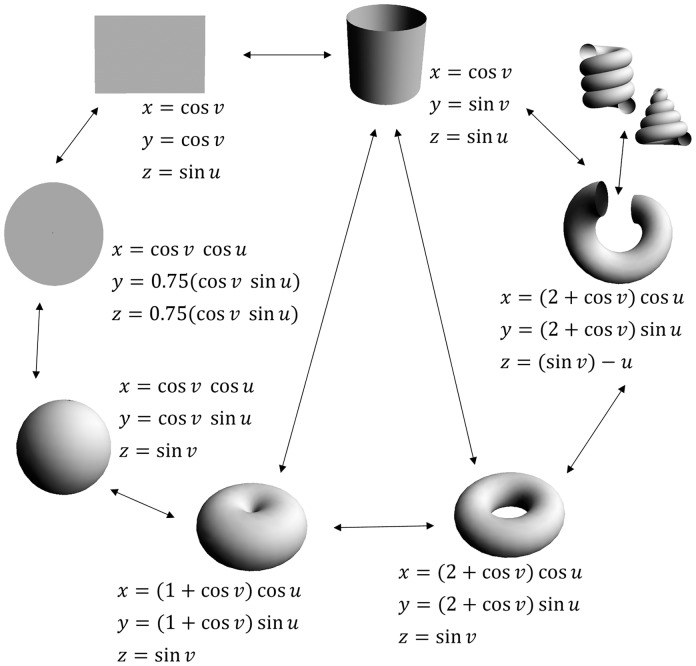
Basic geometric forms are depicted with their sets of parametric 3D equations. Transformations and reverse transformations are given. All forms are expressed as combinations of sines and cosines and 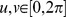.

In general, geometric forms can be parameterized and expressed in terms of particular basic trigonometric functions. Such expressions indicate how basic geometric forms are closely related, and any one of these forms can be a starting point to produce whole 3D forms (Fig. 1). Rather than use helicospiral curves as a starting geometry, as has been done in most previous studies, we instead use a torus as the initial surface and modify it to model a range of whole 3D surfaces. The general form of the organisms of interest ultimately determines which basic geometric form is most appropriate, and our choice of the torus does not preclude using other basic geometric forms as a starting point (Fig. 1). As a regular surface, the torus is expressed as a differentiable function. It can be represented by three parameterized variables given as *x*(*u*, *v*)  =  (*R* + *r* cos *v*) cos *u*, *y* (*u*, v)  =  (*R* + *r* cos *v*) sin *u*, *z*  =  *r* sin *v*, where *R* is the major (outer) radius of the torus horizontally and *r* is the minor (inner) radius of the cross-section of the torus; the parameters are 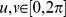 with *u* defining the angle between *R* and the *x*-axis, and *v* defining the angle between *R* and *r* [33].

For gastropod shells, an apical view (i.e., looking at a shell from its top) is generally circular with an offset end, and that end – the aperture – is a cross-section of a tube. The simplest closed-form tube is a torus. The simplest cut is a circular cross-section or meridian, and a cross-sectionally cut torus is used to expose one end to make a coil which can be viewed as a whorl. Elliptical cuts in the torus are produced via Villarceau [34] circles. One end of the cut torus is fixed and the other end is freely suspended, and if we pull downward on the free end, the curvature and torsion of what is now a spiral whorl will change. A coil of *n*-whorls can be produced by extending the length of the tube. The perpendicular of each whorl is also the radius of the cut torus tube, and every whorl has its own coiling axis. Different shapes and sizes of each whorl can be achieved as a result of changes in the geodesic properties (i.e., parallels and meridians) of the cut torus. Aperture and whorl diameters can change at any stage in the coiling process. As the whorls and apertures change in size, they will come in contact with each other as a result of their expansion or contraction. The cut torus may become a cylindrical or conical spiral (Fig. 1, upper right). In general, the whole 3D form is made by the interaction of all aspects of the geodesic properties of the torus.

The torus has geodesic properties on its surface [35]. Lines of curvature on a torus are meridians (lines that create cross-sectional, or poroidal circles) and parallels (lines of latitude with boundaries as outer circles, or toroidals) [36]. Parallels and meridians in *x*, *y*, *z* are orthogonal, and all meridians are of constant diameter, while all parallels are of variable diameter. All meridians and parallels, except for the maximum sized parallel, are geodesics. Tangent vectors of meridians and parallels are principal curvatures [36].

Curvature is the rate of change of tangents given as first derivatives that are perpendicular to each other on the surface [36]–[37]. The first derivatives are lines of curvature on a surface that are curves with tangential points along a principal curvature [38]. For a torus, the principal curvatures are 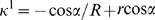 and 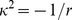 of some angle 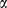 with major and minor radii *R* and *r*, respectively [35]. Torsion can be defined as a point along a curve that can move in different directions and be determined along any of the *n*-space curves of a given surface. Torsion depends on the rate at which a curve is turning along its arc length. On a surface, torsion is definable only for curves with 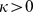.

#### 1.2.1. Moving reference frames

At each point on the surface of a torus, an orthonormal frame consisting of three vectors can be defined. A moving reference frame with respect to velocity and acceleration of that frame occurs at each point along any curve on the surface where the frame is differentiable, and the result is a complete description of change in curvature and torsion. A moving reference frame on the whole torus can be a Darboux frame [39] which is a generalization of a Serret-Frenet [40]–[41] frame.

A Serret-Frenet frame consists of the unit tangent vector,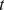, the principal unit normal, **n**, and unit binormal, **b** at a point, *p*, along a curve. These vectors comprise an orthonormal frame, {**t**, **n**, **b**} [37]. In general, **b** is a multiple of **n**, and torsion is a factor in **b** and **n**, while curvature is a factor in **t** and **n** [38]. A Serret-Frenet frame parameterized by arc length, *s*, with velocity vector, **w**, is 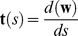, 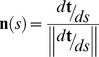, 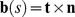 with first derivatives 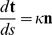, 
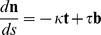
, and 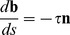. For curvature with respect to *s*, 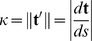
and for torsion, 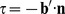. If 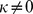, 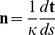 and 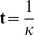; **n** and **t** are perpendicular [37], [42].

**Figure 2:**
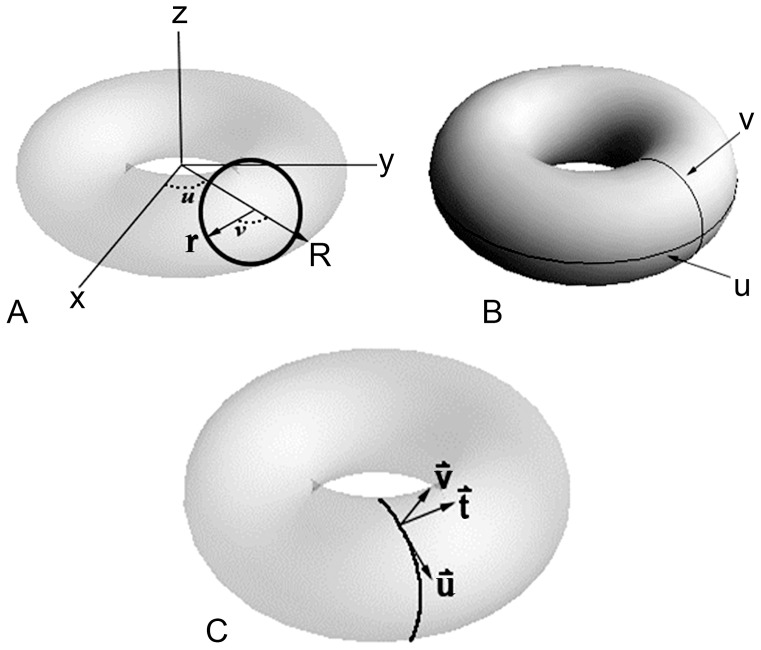
The torus as a model depicted with a Darboux frame. A, A torus in *x*, *y*, *z* space with parameters *u* and *v*, *R* is the radius of a whorl and *r* is the radius of the aperture; B, A torus with *u* and *v* indicated with respect to major and minor radii, respectively; C, A torus with a Darboux frame consisting of a unit tangent vector (**t**), a unit normal vector (**u**), and a tangent normal vector (**v**).

A Darboux frame with respect to *s* has the vectors 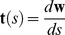, **u**(*s*)  =  **u**(**w**), and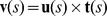, where **w** is the velocity vector, **t**(*s*) is the unit tangent vector as it is in a Serret-Frenet frame, **u** is the unit normal vector, and **v** is tangent normal vector (Fig. 2). Essentially, a Darboux frame is an orthonormal frame where the unit tangent and tangent normal vectors are two principal directions and are linearly independent; **u** and **v** are in tangent planes that are perpendicular to **t** [43]. Since a Darboux frame is a moving reference frame 
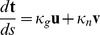
, 
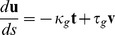
, 
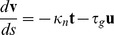
, where 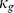 is the geodesic curvature, and 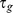 is the geodesic torsion [36].

A Darboux frame exists at any non-umbilic point on a surface. An umbilic point occurs on a surface where the principal curvatures are equal and every tangent direction is a principal direction [39]; i.e., curvature is the same in any direction. Since a torus has no umbilics, and lines of curvature are parallels and meridians [43], Darboux frames exist on all points on a torus. Moreover, with parallels and meridians as principal curves, *u* and *v* are parameters defining angles of the principal curves in terms of the major and minor radii of a torus, respectively [36].

From a Darboux vector field for a unit velocity vector [37], the relationship between Serret-Frenet and Darboux frames is determined by 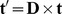, 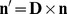, 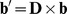, where the Darboux vector field is 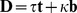
with 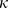
as curvature, 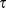
as torsion, and vectors {**t**, **n**, **b**} are a Serret-Frenet frame. For a unit velocity vector with 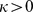 [37], 
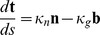
, 
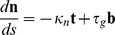
, 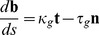, so that the relationship between Serret-Frenet and Darboux frames is evident in terms of curvature and torsion.

#### 1.2.2. Parametric 3D equations

Parametric 3D equations can be used to construct bounded surfaces [44]–[47] such as tori. At each point on the surface of a torus, two parameters, *u* and *v*, and a tangent vector define a Darboux frame (Fig. 2). The torus is a smooth manifold and differentiable everywhere, and parametric 3D equations in *x*, *y*, and *z* are differentiable with respect to parameters *u* and *v*. The derivatives of the vectors of a Darboux frame indicate the behavior of a curve locally on a surface. By modifying the parametric 3D equations for a torus, we construct various models of mollusks and echinoids.

**Figure 3:**
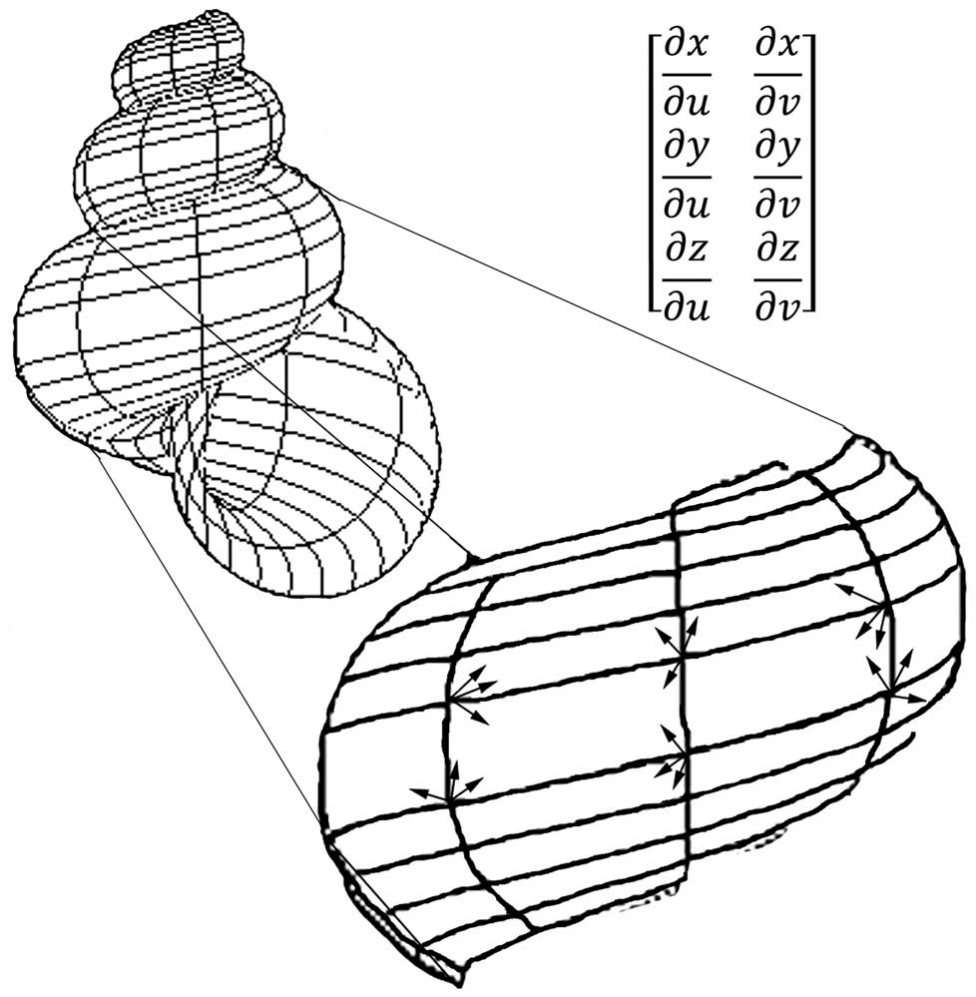
Tangent lines and planes representing Jacobians on a shell model with a gridded surface. A Darboux frame is shown as a trihedron of orthonormal vectors at points on the surface.

First partial derivatives are tangent lines and planes of the whole surface. Numerically, tangent lines and planes at every point on the surface (Fig. 3) are solutions to Jacobian determinants that can also be the elements of the Jacobian matrices (i.e., **Jacobians**). These elements represent degrees of stretching from variables *x*, *y*, *z* to parameters *u*, *v* and represent angular attributes with respect to radii of models constructed. Solutions to the Jacobians are used as variables in principal components analysis (PCA) to create a morphospace. Euclidean distances can be found as the change in tangents of a curve between two points, and because tangents are slopes of the curve, a morphological *surface space* can be constructed based on the Jacobians. The Jacobians are a mathematical summary of the entire surface and present a powerful new way of comparing phenotypes.

## Methods

### 2.1. Parametric 3D Equations

**Figure 4:**
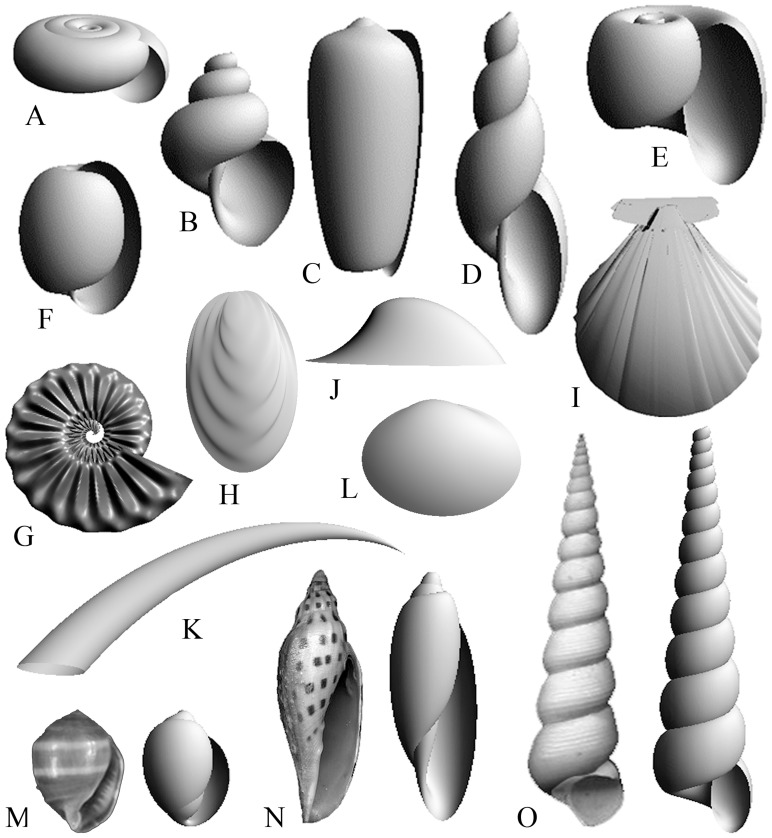
Illustrations of mollusk shell models created from parametric 3D equations. A, System 1 gastropod shell 1; B, System 1 gastropod shell 2; C, cone shell; D, System 1 gastropod shell 3; E, System 2 gastropod shell; F, bubble shell; G, ammonite; H, oyster; I, scallop; J, limpet; K, scaphopod; L, clam. Pictures of gastropod shells and their models: M, *Melampus coffeus*; N, *Scaphella junonia*; O, *Turritella* sp.

All systems of parametric 3D equations are derived from the generalized set that is



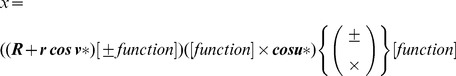
(2.1.1)




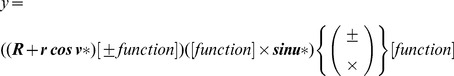
(2.1.2)




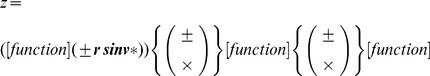
(2.1.3) where emboldened, italicized terms and operators represent a torus, asterisked variables can have coefficients, and multiple operators are possible with respect to other functions. The label, [*function*], can be a value multiplied by 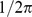, an exponential with respect to 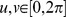, a sine, cosine, hyperbolic tangent, hyperbolic secant, or some combination. Some of the models are depicted in Figure 4. Parametric 3D equations used to construct gastropod shells (with the exception of limpets) are identified with the term “System” and model number.

Specimen pictures [48], [49] (e.g., Fig. 4M, N) used for comparison to gastropod shell models and measurements of *R_max_* and *r_max_* are listed in Table 1. Measurements were made from pictures [48], [49] and verified using specimens from the University of Michigan Museum of Zoology (UMMZ) (Tables 1 and 2). Scientific names are accepted in the World Register of Marine Species (WoRMS; www.marinespecies.org) and Integrated Taxonomic Information System (IT IS; www.itis.gov) databases. *R_max_* and *r_max_* are measured, and the nearest integer values are reduced to the lowest possible ratio.

**Table 1:** Measurement of maximum whorl and aperture radii (*R_max_* and *r_max_*) of gastropod shells.

Mollusk shell example	Actual measurement (cm)		Actual proportion		Integer proportion	
	*R*_max_	*r*_max_	*R*_max_	*r*_max_	*R*_max_	*r*_max_
*Scaphander lignarius*	1	1.5	1	1.5	1	2
*Sinum perspectivum*	1.6	1.6	1	1	1	1
*Melampus coffeus*	0.7	0.85	1	1.214	1	1
*Oliva sayana*	0.8	1.35	1	1.687	1	2
*Longchaeus candidus*	0.55	0.45	1.222	1	1	1
*Lithopoma phoebium*	1.8	0.75	2.4	1	2	1
*Scalenostoma subulatum*	0.55	0.4	1.375	1	1	1
*Architectonica nobilis*	1.45	0.4	3.625	1	4	1
*Heliacus variegatus*	0.8	0.3	2.667	1	3	1
*Calliostoma bairdi*	1.2	0.45	2.7	1	3	1
*Janthina globosa*	1.35	1.25	1.08	1	1	1
*Vitrinella oldroydi*	0.7	0.25	2.8	1	3	1
*Scaphella junonia*	0.9	1.55	1	1.722	1	2
*Buccinum glaciale*	1.4	1.05	1.333	1	1	1

**Table 2:** Material examined from the University of Michigan Museum of Zoology (UMMZ).

Name of Taxon; UMMZ Number; Locality and Collector Information.	
*Scaphander lignarius* (Linnaeus, 1758); UMMZ 9423; England.	
*Sinum perspectivum* (Say 1831); UMMZ 19526; Bahamas.	
*Melampus coffeus* (Linnaeus, 1758); UMMZ 236294; Bird Key, NR., St. Petersburg, Pinellas Co., Florida; coll.: J.B. Clark.	
*Oliva sayana* Ravenel, 1834; UMMZ 251580; Big Gaspilla Island, Florida; coll: Carl Fellons, 3/62.	
*Longchaeus candidus* (Mörch, 1875); UMMZ 165937; Grassy Key, Florida Keys; coll.: B.R. Bales.	
*Lithopoma phoebium* (Röding, 1798); UMMZ 198132; McGinty's Ocean Ridge, Florida; coll: M.M. Solem.	
*Scalenostoma subulatum* (Broderip, 1832); UMMZ 19940; Mauritius.	
*Architectonica nobilis* Röding, 1798; UMMZ 250913; Florida, Gulf of Mexico; dredged.	
*Heliacus variegatus* (Gmelin, 1791); UMMZ 250920; Hawaii.	
*Calliostoma bairdi* Verrill & Smith, 1880; UMMZ 250829; Florida.	
*Janthina globosa* Swainson, 1822; UMMZ 18272; Japan.	
*Vitrinella oldroydi* Bartsch, 1907; UMMZ 174914; Monterey, Pacific Grove, California; coll: Chase 1941.	
*Scaphella junonia* (Lamarck, 1804); UMMZ 251654; 20 fms., off Cameron, Louisiana.	
*Buccinum glaciale* Linnaeus, 1761; UMMZ 17166; Greenland.	

### 2.2. Jacobians

The following example using a set of parametric 3D equations for a ring torus illustrates how the surface is represented by a Jacobian. Let the equations for a ring torus be *x*  =  (1+ cos *v*) cos *u*, *y*  =  (1+ cos *v*) sin *u*, *z*  =  sin *v*, where *R*  =  *r* = 1 and 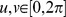. From Jacobian determinants of the mapping *x*  =  *f* (*u*, *v* ), *y*  =  *g* (*u*, *v* ), *z*  =  *h* (*u*, *v* ), the Jacobian (i.e., Jacobian matrix) evaluated at *u*  =  *v* = 0 is


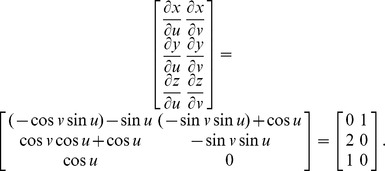
2.1.4

#### 2.2.1. Relationship between parametric 3D equations and Jacobians

A surface is implicitly defined as *x  =  f*(*u*, *v*), *y  =  g*(*u*, *v*), *z  =  h*(*u*, *v*) with differentials that form tangents [50]. The derivative of a parametric function is a velocity vector since tangents to a curve are the speeds at which a point on the curve moves.

Partial derivatives for *x*, *y*, and *z* with respect to *u* and *v* via the general chain rule [50] are


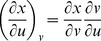
 and 
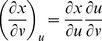
 for differential 
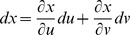
,
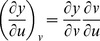
 and 
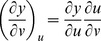
 for differential 
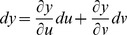
,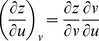 and 
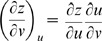
 for differential 
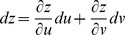
.

Define three implicit functions [49], [51] of the surface as *F*(*x*, *y*, *z*)  =  *F*[*f*(*u*, *v*), *g*(*u*, *v*), *h*(*u*, *v*)],

*G*(*x*, *y*, *z*)  =  *G*[*f*(*u*, *v*), *g*(*u*, *v*), *h*(*u*, *v*)], and *H*(*x*, *y*, *z*)  =  *H*[*f*(*u*, *v*), *g*(*u*, *v*), *h*(*u*, *v*)] that describe the relation among *x*, *y*, *z* with respect to *u* and *v*. The differentials for *F*, *G*, and *H* in terms of *x*, *y*, *z* are 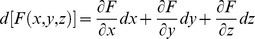, 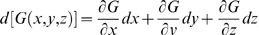, 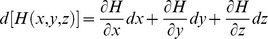, respectively, where *dx*, *dy*, and *dz* in terms of *u* and *v* can be substituted into each equation in this set of linear equations, and *F*, *G*, *H* are tangent planes that define the intersecting surfaces of a model.

Let *F*(*u*,*v*)  =  *f*[*x*(*u*, *v*), *y*(*u*, *v*), *z*(*u*, *v*)], *G*(*u*,*v*)  =  *g*[*x*(*u*, *v*), *y*(*u*, *v*), *z*(*u*, *v*)], and *H*(*u*,*v*)  =  *h*[*x*(*u*, *v*), *y*(*u*, *v*), *z*(*u*, *v*)] be defined as implicit functions. The differentials for *F*, *G*, and *H* in terms of *u* and *v* are 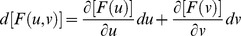, 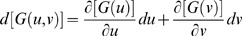, 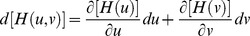. The relation between *x*, *y*, *z* and *u*, *v* with respect to differentials for *F* is 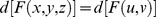. For 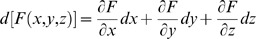, and substituting equations for *dx*, *dy*, *dz* in terms of *u* and *v* gives
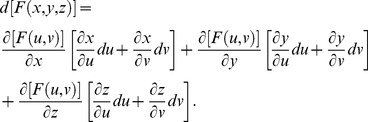



By gathering terms and using the chain rule, 
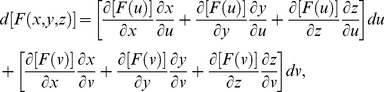
there fore, 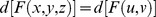. For *G* and *H*, the same treatment will yield 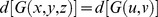 and 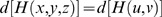, respectively. The total differential indicates the relation among *x*, *y*, *z* with respect to *u* and *v* for intersecting tangent planes in terms of *F*, *G*, and *H*. In general, three dimensions (*x*, *y*, *z*) are mapped into two dimensions (*u*, *v*), and any coordinate system can be used [50], [52].

Intersecting tangent planes represent the surface of a model. In vector notation, the normal planes of the models are 
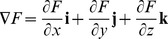
, 
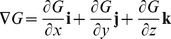
, 
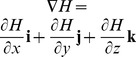
, where 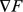, 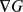, and 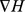 are gradients of *F*, *G*, and *H*, respectively.

A surface is a system of linear functions in *dx*, *dy*, *dz* with respect to differentials of implicit functions *F*, *G*, *H*. The Jacobian determinants give the best linear approximations [49] to the differentiable functions *dx*, *dy*, *dz*, and they are the generalized gradients in terms of *F*, *G*, *H*. Now, we can solve Jacobian determinants of implicit functions, *F*, *G*, *H* with respect to differentials *dx*, *dy*, *dz* in terms of functions *f*, *g*, *h* with respect to differentials *du*, *dv* that represent the surfaces of models as tangent planes and tangent lines in a form that is amenable to simpler calculation.

Implicit equations for *F*, *G*, *H* where variables defined as *x*, *y*, *z*, *u*, *v* are 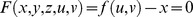, 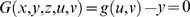, 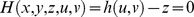,and the differentials are 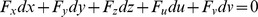, 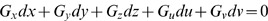, 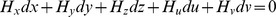. For differentials *du* and *dv* with respect to *f*, *g*, *h*, the vector



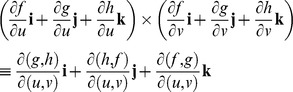
is normal to a point, *p*, on the surface of the model [50].

From Cramer's Rule [50], [52], solutions to the partial derivatives with respect to *F*, *G*, *H* are


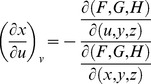
 and 
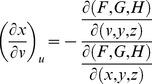



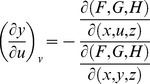
 and 
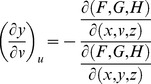



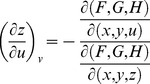
 and 
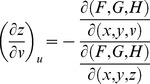
.

The partial derivatives are expressed in terms of the Jacobian determinants with 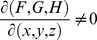. Each partial derivative of *x*, *y*, *z* with respect to *u*, *v* is an element of the Jacobian matrix used to represent the surface of a model.

## Results

### 3.1. Solutions to the Generalized System of Parametric 3D Equations

Analytically, parametric 3D equations allow for flexibility in producing a wide range of 3D forms. In our examples, the protocol has been to make generalized 3D models of planispiral and conispiral gastropods, bivalves (unwound forms), and rounded echinoids. The parametric 3D equations that describe surface models explicitly include the values for maximum whorl and aperture radii (*R_max_* and *r_max_*, respectively) for mollusk shells and maximum radii (*R_max_* and *r_max_* are approximately equal) for echinoids. Following the generalized set of parametric 3D equations (Section 2.1.), generalized models may be produced based on *R_max_* and *r_max_* obtained from pictures of actual shells or specimens. These generalized forms serve as morphological “blanks” that, when subjected to refinements via further measurements and reverse engineering, can be quite realistic and dimensionally accurate. Refinements entail experimenting with coefficient values and iteratively comparing the resulting model with measurements made from real shells until an acceptable degree of similarity has been obtained.

In all cases,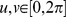. For gastropod morphologies, three systems of equations are developed, one of which is for limpets. The first gastropod shell generating system (System 1) (e.g., Figs. 4A, B, C, D, F) is



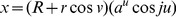
(3.1.1)




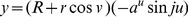
(3.1.2)




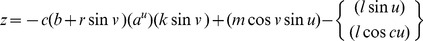
(3.1.3)where coefficients are 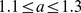, 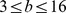, 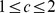, 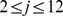, 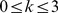, 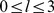, 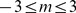, 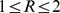, and 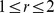. Special cases highlighted in our study include normally coiled turritellids (e.g., Fig. 4O) and spirals as



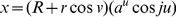
(3.1.4)




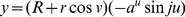
(3.1.5)




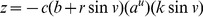
(3.1.6)


*Vermicularia* as



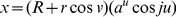
(3.1.7)




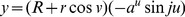
(3.1.8)




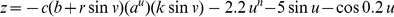
(3.1.9)where coefficients are defined by those in System 1 equations with the exceptions of 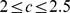
and 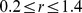, and an additional coefficient is 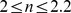
for *Vermicularia*. For *R_max_*  =  *r_max_*  = 1, high values of *b*, and 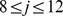, normally coiled turritellid shells result. *Vermicularia* start out as a normal turritellid shell, but negative values of *b* and a squared term in the *z*-direction produce the unwound bottom half of the shells. Spirals require that *r_max_*approaches but is not equal to zero, making a “curve” with the smallest possible cross-section.

The second gastropod shell generating system (System 2) (e.g., Fig. 4E) is



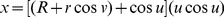
(3.1.10)




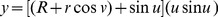
(3.1.11)




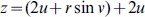
(3.1.12)where 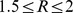 and 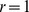.

A system of equations for limpets (e.g., Fig. 4J) is



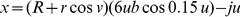
(3.1.13)




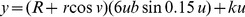
(3.1.14)




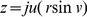
(3.1.15)where 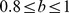, 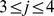, 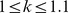, 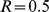 and 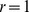.

For Systems 1 and 2, values for coefficients and *R_max_* and *r_max_* vary within narrow ranges. Coefficients *a* and *c* each partially affect overall shell width and height with respect to the *x*-, *y*-, *z*-directions. Coefficients *b* and *j* each partially affect overall height with respect to number of whorls in the *z*-direction. Coefficients *k*, *l*, and *m* affect overall shape of the shell in terms of changes in whorl radius with respect to the *z*-direction.

Specific values for *R_max_* and *r_max_* and coefficients are necessary to produce specific shells. When *k* = 0.7, *l*  =  *m* = 3, *c* = 1, *R_max_*  = 1.5, and *r_max_*  = 2, a theoretical cone shell is created. For *l* = 1 or 2 with respect to a sine function, *k* sin *v* = 1, *m* = 0, *R_max_*  = 1, and *r_max_*  = 2, theoretical bubble shells are represented.

Systems of parametric 3D equations were developed for exemplar forms of bivalves, cephalopods, and scaphopods. For bivalves, three systems are based on the following: for clams (e.g., Fig. 4L),



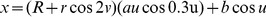
(3.1.16)




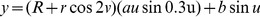
(3.1.17)




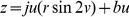
(3.1.18)where 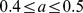, 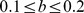, 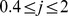, *R*  =  *r* = 1; for oysters (e.g., Fig. 4H),



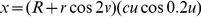
(3.1.19)




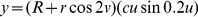
(3.1.20)




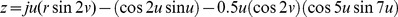
(3.1.21)where 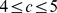 and *R*  =  *r* = 1; for scallops (e.g., Fig. 4I),



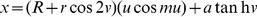
(3.1.22)




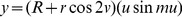
(3.1.23)




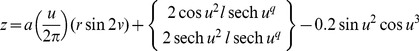
(3.1.24)where 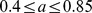, 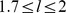, 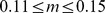, 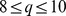, 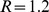, and 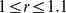.

For bivalves, coefficient *a* controls the presence and shape of the auricles for scallops with respect to the *x*- and *y*-directions. Coefficient *b* defines the position of the umbo in clams with respect to the *z*-direction. Coefficient *c* is important for oysters in terms of length and shape (degree of concavity or roundness) of the shell with respect to the *x*- and *y*-directions, while for clams, length is controlled by coefficient *j* with respect to the *z*-direction. Shape for scallops is influenced by coefficient *l* in the *z*-direction and coefficient *m* in the *x*- and *y*- directions in terms of width and degree of flatness, respectively.

For cephalopods (e.g., Fig. 4G) a system of parametric 3D equations for ammonites is



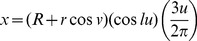
(3.1.25)




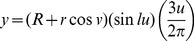
(3.1.26)




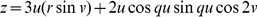
(3.1.27)where 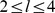, 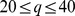, 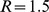, and *r* = 1. In the *x*- and *y*- directions, degree of coiling is controlled by coefficient *l*. The degree of folding or pleating is defined by coefficient *q* with respect to the *z*-direction.

The system of equations representing scaphopods (Fig. 4K) is



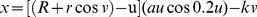
(3.1.28)




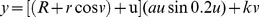
(3.1.29)




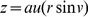
(3.1.30)where 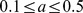, 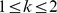, 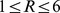, and 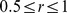. Coefficient *a* controls length and degree of bending with respect to the *x-*, *y-*, *z*-directions, while coefficient *k* influences the overall width with respect to the *x*- and *y*- directions.

Aside from changes in coefficient values, changes in *R* and *r*, when considered in tandem, reveal the extent that each variable – *x*, *y*, *z* – influences changes in modeled shell morphologies. If *R* changes and *r* is held constant, modeled shells will widen (coiled gastropods), change in symmetry (limpets), lengthen and become rounder (bivalves), become narrower (ammonites), or become flatter and narrower (scaphopods) with respect to *x* and *y*. If *R* is held constant and *r* changes, modeled shells will become wider, rounder and elongated (coiled gastropods), flatter and elongated (limpets), wider (clams, oysters and scaphopods), wider and rounder (scallops), or wider and have whorls that merge (ammonites) with respect to the *z*-direction.

Modeled counterparts to actual specimens were constructed to show the efficacy of using *R_max_* and *r_max_* in representing these taxa. Along with these values, a small range of specific values for coefficients is used. For example, when *k* = 1, *l*  =  *m* = 0, *R_max_*  = 1.5, and *r_max_*  = 2, the result is a volute (e.g., Fig. 4N). For *l* = 2, *k* = 1, or 2 with respect to a cosine function, *m* = 0, *R_max_*  = 1, and *r_max_*  = 2, an olive shell is created. When *l* = 1.5, *m* = 2.3, *R_max_*  = 0.7, and *r_max_*  = 2.4, a *Melampus* is made (e.g., Fig. 4M). To make a sundial shell, *R_max_*  = 1.3, and *r_max_*  = 1, *l* sin *u* = *l* cos *cu*  = 0 with *a*  =  *b* = 2.

**Figure 5:**
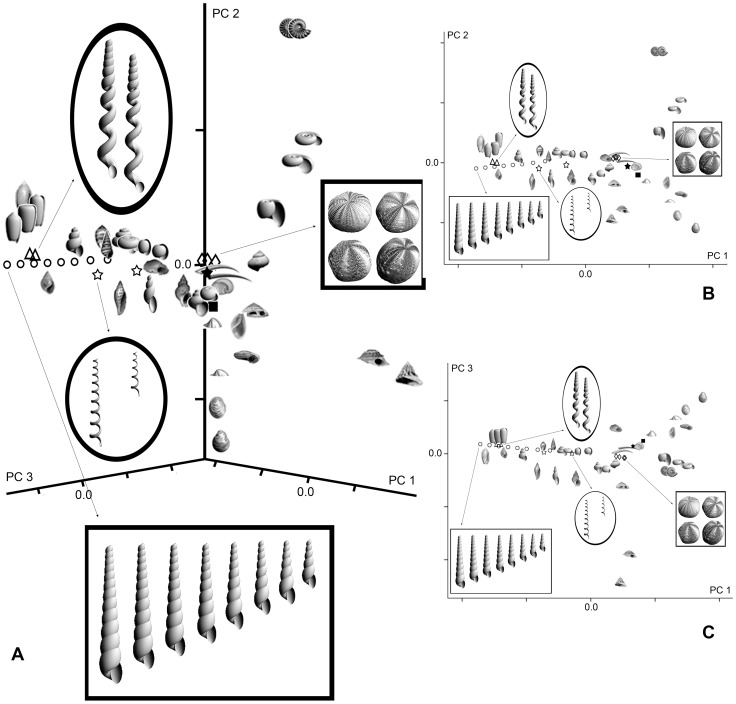
A, PCA ordination of modeled and actual mollusk shells as well as echinoid models and spiral curves in morphospace; ? =  modeled clams; •  =  modeled scallops. Insets depicted are ▵  =  *Vermicularia* (upper left), ?  =  spirals (middle left), ?  =  isometric turritellid series (lower center), ◊  =  echinoids (upper right). Models in each inset match symbol order in the ordination from left to right; B, PC2 vs. PC1; C, PC3 vs. PC1.

Finally, as a contrast to mollusk shells, a system of parametric 3D equations for echinoids was devised. Just as was done with mollusks, we start with a torus and only use sine, cosine, hyperbolic secant and hyperbolic tangent functions to produce the desired forms. The result is the system of equations given as



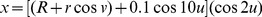
(3.1.31)




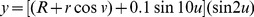
(3.1.32)




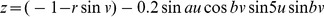
(3.1.33)where 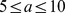, 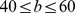, 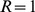, and *r* = 2. Negative terms are used in the *z*-direction with the last term being a polynomial in sines and cosines, and the hole in the starting torus almost disappears so that the resultant form is approximately spheroid to ellipsoid (see inset-Fig. 5).

### 3.2. Jacobians and Interpretation of Mollusk and Echinoid Forms

The majority of the forms modeled are mollusk shells. In the Jacobians, the first column of elements is associated with whorl attributes. In the *x*-direction, small changes in whorl radii are generally associated with a high positive value for the first element, and in the *y*-direction, tightness of coiling is associated with a high negative value for the second element. In the *z*-direction, forms with a higher spire tend to have larger negative values for the third element and low spired to planispiral forms tend to have large positive values. The second column of the Jacobians is associated with aperture attributes. The highest positive values in the *x*-direction are associated with forms that have relatively large apertures. The highest negative values in the *y*- and *z*-directions are associated with irregular and elongated apertures, respectively. For echinoids, the Jacobians are mostly defined by a large positive value for the second element of the first column and a low negative value in the third element in the second column of values. These values reflect the roundness of echinoid forms.

### 3.3. Interpretation of Mollusk and Echinoid Surface Morphospace

The numerical solutions of the Jacobians were used as variables in a principal components analysis (PCA) to produce a morphospace (Fig. 5A– 3D PCA; Fig. 5B– PC2 vs. PC1; Fig. 5C– PC3 vs. PC1). Our use of Jacobians ordinated in an empirical morphospace differs from purely theoretical approaches in which the morphological axes are based directly on the underlying mathematical model. The empirical spaces generated with our method contain a combination of purely theoretical shells and empirically based forms. Whether using empirical or theoretical axes in morphospace construction, the representation of variation among whole 3D surfaces in our morphospace is depicted with independent axes.

From PCA, eigenvalues for the first six PCs are 2.042, 1.183, 1.037, 0.820, 0.566, and 0.352, and the percent variance explained is 34.029, 19.722, 17.275, 13.664, 9.435, and 5.874, respectively. The percent cumulative variance is 53.751 for the first two PCs, 71.027 for the first three PCs, 84.691 for the first four PCs, and 94.126 for the first five PCs.

The highest correlations of Jacobian elements with PCs are: PC1 with 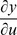
(0.844); PC2 with 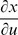
(0.840); PC3 with 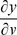
(0.896); PC 4 with 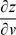
(0.647); PCs 5 and 6 have correlations that are <0.50. On PC1, the next highest correlation occurs with 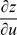
(0.693) and 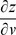
(0.694), while for PC2, the next highest correlation is associated with 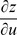
(−0.608) as well.

For modeled mollusk shells, partial derivatives in the *z*-direction reflect changes in aperture radii as each whorl radius changes. Partial derivatives with respect to *u* (whorls) in the *x* and *y*-directions have more influence on overall modeled shell morphologies than partial derivatives with respect to *v* (apertures). Jacobian elements,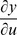, 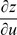, and 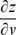, that are highly correlated to PC1, indicate tightness of coiling and relative aperture elongation. Whorl and aperture dimensions relative to each other are indicated by high correlation of Jacobian elements 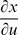, and 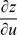 with PC2. PC3 is highly correlated with Jacobian element 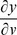, which indicates aperture shape.

In general, the morphospace for modeled mollusk shells can be divided roughly in terms of the relationship between maximum whorl (*R_max_*) and aperture radii (*r_max_*). Small changes in whorl radii of high spired shells with small rounded to elongated apertures are represented by 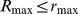 (Table 1). Large changes in whorl radii of low spired shells with irregularly rounded to elliptical apertures are represented by 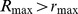 (Table 1). In the morphospace, high-spired mollusk models (i.e., those with spirals that are stretched in space) group together extending into gradients as do planispiral to lower spired mollusk forms (i.e., those with spirals in or nearly in the plane). Unwound and low-spired mollusk models produce gradients that converge near the rounded echinoid forms. The insets (middle left and lower center) in Figure 5 illustrate ontogenetic trajectories for simulated turitellid models. Isometric accretionary growth is depicted by a linear trajectory of a turritellid having from five to twelve whorls. The modeled *Vermicularia* have the same trajectory as a normally coiled turritellid until growth becomes allometric, and the whorls begin to separate [53]. Theoretical spiral elements also illustrate a separate “ontogenetic” trajectory (Fig. 5) and show how the method can be used to study basic spiral geometry.

The normally coiled turritellid series represents an ontogenetic trajectory illustrating isometric growth, with changes in number of whorls while the overall proportions of each whorl remain the same. The contrast between a normal turritellid and the *Vermicularia* illustrates allometry. The normal turritellid trajectory is a sequence of whorls numbering five (closest to the PC2 axis) to twelve, paralleling PC3 (Fig. 5). Modeled *Vermicularia* with nine whorls exhibit different degrees of uncoiling and occur at a slight angle from the normal turritellid with nine whorls (Fig. 5). In close proximity a parallel trajectory of spiral curves (with infinitesimal diameters) are plotted with five and nine coils, respectively, and occur below the turritellid sequence.

Each PC is correlated with Jacobian elements related to whorl or aperture characteristics for mollusk shells or spheroidal-ellipsoidal features when considering echinoids. For mollusk shells, PC1 was highly correlated with elements related to the tightness of coiling and relative elongation of the aperture. PC2 was most highly correlated with elements related to relative whorl and aperture dimensions. PC3 had a high correlation with elements related to aperture shape. For echinoids, PC1 defined degree of elongation, while PC2 defined breadth, and PC3 defined overall shape.

Jacobian elements with respect to *u* (PCs 1 and 2) for normal turritellids, *Vermicularia*, and spiral curves represent coiling attributes. In particular, 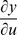
(PC1) indicates degree of coiling (i.e., number of coils), and 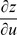
(PC1) indicates tightness of coiling. For 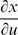
(PC2), height of spire is indicated. In addition, changes in 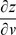
(PC1) indicate degree of change in aperture size from a normal turritellid or a *Vermicularia* form to the infinitesimal diameter of a spiral curve. As with modeled mollusk shells in general, 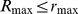.

Modeled echinoid morphologies are spheroidal and ellipsoidal forms that occupy morphospace near PC2 (Fig. 5). Each element of the Jacobian recovers test shape as a closed form. The Jacobian element 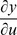 is the predominant influence (PC1) with 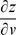 a secondary influence (PC1 and PC4) as changes in the *z*-direction overtake changes in the *y*-direction with respect to a spheroidal or ellipsoidal shape. For echinoids, 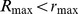.

For models where tangent lines and planes fall into the same plane, an interesting result emerged. The two ammonites that differ very slightly morphologically had identical Jacobians and plot in the same location in morphospace (Fig. 5; to allow for visibility, the ammonite models are jittered). One model exhibits a shift in a repeated sine wave (i.e., pleating) parallel to and shifted in the direction of *v* with respect to the other model (Figs. 4G and 5), producing indistinguishable Jacobians. In the second model, a shift in pleats parallel to and in the direction of *u*, produced Jacobians that were different but close in value. For the first case, a post-processing step related to Jacobians can be used numerically to produce morphological differences. More information and recommendations are given below.

### 3.4. Heuristic Tests of the Meaning of Surface Morphospace

**Figure 6:**
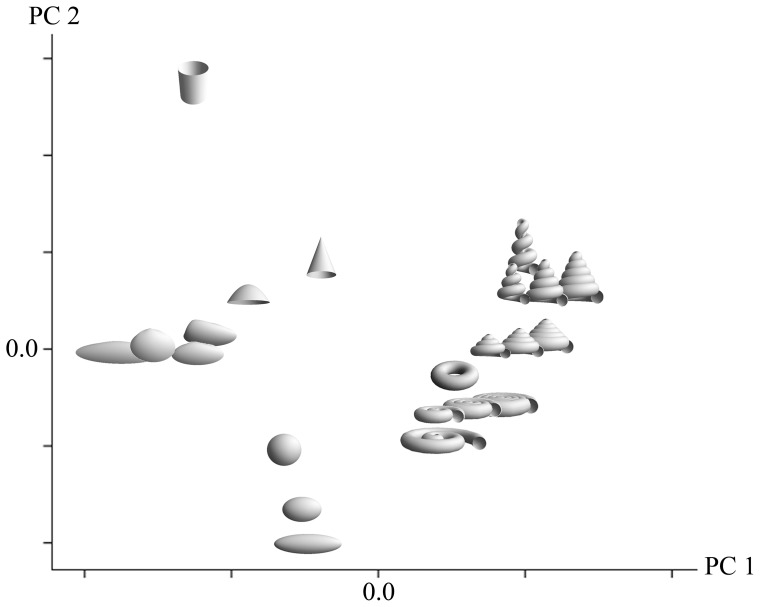
PCA of basic geometric forms and select mollusks to heuristically explore the properties of surface morphospaces based on Jacobians.

The use of PCA in our method means that the axes of the resulting morphospace are independent. However, each axis represents both parameters *u* and *v,* and interpretation of the axes requires consideration of each parameter changing relative to the other, potentially making axis interpretation more complicated. To better understand the meaning of the axes and the properties of surface morphospaces based on Jacobians, 3D models of familiar basic geometric forms were created and their surfaces ordinated using PCA (Fig. 6). In addition, a limited number of bivalve and gastropod models, including slight variations, were generated to determine how these forms plot relative to one another. For basic geometric forms we used a torus, cylinder, cone, dome, sphere, and two ellipsoids in which the lengths of the major axis differ. For bivalves we generated models showing variation in the shape of the outline as well as some differences in the way the umbones coil. Gastropods with forms ranging from planispiral to conispiral and varying in whorl number were also included. With the exception of ellipsoids, we have not conducted controlled heuristic experiments in which one aspect of morphology is varied and all others held constant (but, see turritellid ontogenetic trajectory in Figure 5, where only whorl number is varied). Rather, our heuristic tests are intended simply to determine if ordination based on Jacobians succeeds in capturing the major differences in surface geometry among a smaller number of basic forms. The morphospaces generated using PCA are empirical, and therefore, not directly comparable. However, there are similarities in the models used in this heuristic test and the mollusk-echinoid morphospace shown earlier (Fig. 5). The tests should, therefore, be useful for interpretation of each space and for evaluating the method more generally.

The resulting PCA (Fig. 6) very effectively ordinates forms based on differences in their surface geometry. Domed, conical, heliconical, toroidal/planar, and spherical/ellipsoidal forms all plot in different regions of the morphospace, and the relative positions of different morphologies reflect the degree of similarity in surface geometry. The dome plots relatively close to the cone and can be viewed as a rounded cone. Although bivalves are rapidly expanding helicones, their overall shape is similar to a dome, and their position near the dome in the morphospace confirms this. Among the coiled forms, there is a general progression from planispiral to more conispiral forms along PC2. Slight variations in the tightness of coiling and increases in the number of whorls within a morphology are captured and form morphoclines along PC1. The torus can be viewed as a planar uncut coil and is nearest the planispiral gastropods. Spheroidal and ellipsoidal forms occur as a morphocline reflecting elongation in the major axis and are roughly equidistant from coiled and uncoiled forms. The cylinder is an uncoiled tube that is located furthest from all other forms.

As was the case for the ordination of mollusks and echinoids, partial derivatives 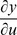, 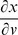, and 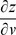
represent number of coils, whorl overlap (i.e., partial aperture shape), and aperture size but, because these are empirical spaces, the correlations with PC1 are slightly different (0.962, −0.679, and 0.899, respectively). Similarly, partial derivatives 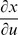, 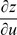, and 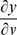 represent height of form, tightness of coiling (or lack thereof) and aperture shape (and to some degree, size) and are correlated with PC2 (−0.669, 0.727, and 0.598, respectively). In contrast to other methods that have been used to model and plot shells, the partial derivatives used here include the number of coils/whorls (from undefined in rounded forms to zero in bivalves to some number for gastropods) and potentially allow for the parsing of other morphological attributes as well.

The results of these simple heuristic tests validate our earlier interpretations of the mollusk-echinoid space and demonstrate that this approach can be useful for analyzing a broad range of surface geometries. Many organisms and structures have overall forms that approximate some of the basic geometries used here and our analysis suggests that this method will effectively permit the ordination of a broad range of biological surfaces as well.

## Discussion

The great diversity of surface morphology that exists makes the study of surfaces a natural extension of the study of phenotypic form. Methods for capturing and manipulating 3D objects are becoming more accessible, and there is growing interest in approaches that make fuller use of 3D data, including the study of surfaces. For many questions, surface morphology is arguably a more complete description of the phenotype compared to methods that rely solely on points, distances between points, or outlines. Our general approach in which the entire surface is modeled and parameterized, summarized numerically with the Jacobian, and ordinated using conventional PCA presents a potentially powerful complement to existing methods for studying morphology more generally and biological surfaces explicitly.

Measurements obtained from actual specimens, pictures in monographs, digital images, or point cloud data can be used when making models. Accuracy and fine morphological differences can be obtained by testing variables, parameters and coefficients of parametric 3D equations in an ordered fashion to determine the effect that one change has on the form being produced. For example, one could change the value of one coefficient while holding all other coefficients constant to determine the effect. Extremely detailed models that may capture individual level variability are possible.

Jacobian solutions capture the behavior locally at each point on the surface. A property of Jacobians is size invariance, thus two identical forms that differ in size only will have identical Jacobians and occupy the same location in morphospace. Specimens with very different shapes and sizes can be jointly analyzed, but because 3D parametric models and their Jacobians exclude size, the resulting ordination will also be size-free and reflect differences in surface form only. Rotation and reflection are also important properties of Jacobians. Rotation is modeled as a switch in parameters in the *x*-, *y*-, or *z*- direction. Reflection (or change in symmetry) is modeled as a change in sign in the *x*-, *y*-, and/or *z*-direction. Because of the properties of size, rotation, and reflection associated with Jacobians, detecting large intragroup and/or intergroup variation is possible.

The Jacobian is capable of capturing morphological variation provided that tangent lines and planes differ among models. For 3D models starting with a torus, the degree to which Jacobians have similar numerical solutions and similar morphologies is affected by similarity in measured *R_max_* and *r_max_* if measurement is in approximately the same plane (i.e., *u* and *v* are approximately in the same plane). If measurement between *R_max_* and *r_max_* is farther apart, Jacobian solutions will be different, describing more disparate morphologies. In cases where 3D surfaces approach 2D planar figures, the Jacobian may not recover such differences numerically, or the differences may be quite small, despite the fact that positional shifts may have significance biologically. If the 3D model is reduced to 2D (i.e., changes in the *z*-direction are not measurable), the Jacobian determinant will be zero (i.e., the tangent lines and planes are equal to zero) and the surface is a plane. At this point, a 2D method (e.g., outline analysis) could be used.

In general, the Jacobian is best suited for curved surfaces. We recommend visually inspecting the models and the Jacobians (or the ordination) to ensure that perceived variation is captured. Alternatively, one could simulate relevant morphological differences and test if the Jacobian is sensitive to these. If a difference is not recordable in the Jacobian, then post-processing by using a Hessian matrix (Hessian) or some of the elements of a Hessian may be calculated and used in morphospace construction.

### 4.1. Parametric 3D Equations, Partial Differentiation, and Jacobians

**Figure 7:**
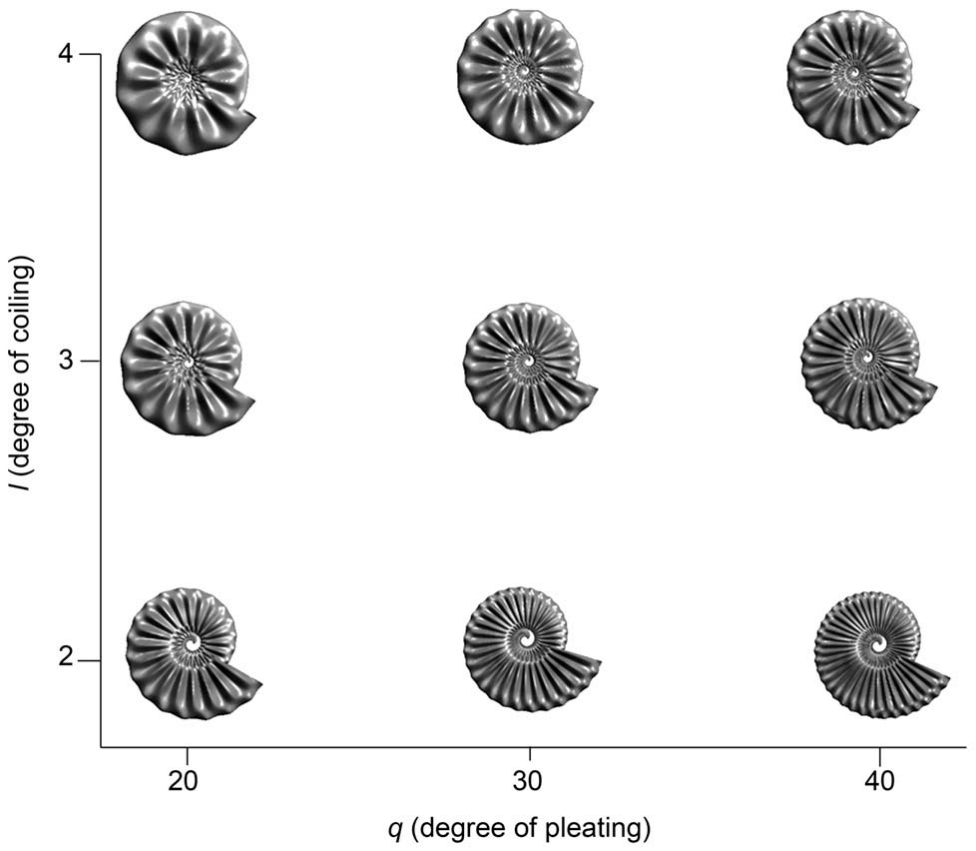
Ammonite shell models with *R* and *r* held constant, showing the result of combinations of coefficient *l* (degree of coiling) in the *x*- and *y*-directions and coefficient *q* (degree of pleating) in the *z*-direction. Coefficient *q* is related to change in aperture (*r* and *v*), and coefficient *l* is related to change in whorls (*R* and *u*).

In our analyses exemplifying the utility of the methods we describe, we use homogeneous equations to find numerical solutions to Jacobians. Boundary conditions on all sets of parametric 3D equations are 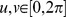. When constructing ammonites, we made incremental changes in one or two coefficients in sets of parametric 3D equations with the intent of producing very similar models. In general, small changes in coefficient and/or radii values can produce large changes in surface morphology from parametric 3D equations, and these differences are definable with respect to partial differential equations. However, if systems of equations used are almost identical to one another, the partial differential equations will have solutions, but those solutions are not necessarily or sufficiently smooth; therefore, Jacobians for very slightly different forms within a given set of parametric 3D equations might have (near-) identical solutions. That is, the tangent lines and planes of very similar surfaces may have solutions to Jacobians that are the same. We use sets of parametric 3D equations that are almost identical in the case of ammonite shells. For example, a difference in the spacing of the pleats (coefficient *q*) – a shift of a sine or cosine – can slightly affect the tightness of coiling (coefficient *l*) (Fig. 7). Using the same set of parametric 3D equations with very slight changes, the model differences just described occur only by slight stretching of the same basic form in one direction, or addition or subtraction of whorls overlapping in the same plane. In addition, there is a shift with respect to *v* from one model to the other. This is like a phase shift for the same tangent lines and planes. The result is the same solutions to the Jacobians occur for both models (Figs. 4G and 5) used in the morphospace.

With tangent lines and planes being the same in the *z*-direction with the *x*- and *y*- directions remaining the same, slight modifications of parametric 3D equations in the *z*-direction will produce different solutions to Jacobians for surface models that are nearly identical. In the case of the ammonites (Figs. 4G and 5), change from one model to the next can be used with respect to *v*, so this parameter is added to the *z*-direction in one of the models. That is, a new set of parametric 3D equations is created. The new model that results is the same as the previous one since the model adds a value of *v* that affects all of the *z*-direction. For one of the ammonite models, the addition is *v* = −1.

Alternatively, imposing the additional constraint of post-processing by calculating Hessian or partial Hessian matrices can be used to produce differences among forms, regardless of the behavior of the Jacobians for all models considered. For example, we did calculations for some of the elements of Hessians for some of the ammonite forms depicted in Figure 7. The elements of Hessians are second partial derivatives, including iterated and mixed partial derivatives. Jacobians have elements that are first partial derivatives. The same number of Hessian elements is calculated for each of the forms. Solutions to the Hessians produced different values for comparable elements in contrast to their corresponding Jacobian elements. Six Hessian elements were calculated, and differences were evident in second partial derivatives 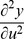 and 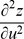 for the ammonites.

Jacobians represent a linearization near critical points, and Hessians (or partial Hessians) represent critical points or extrema on surfaces, and therefore are descriptors of surface features (local maxima and minima). Hessian determinants represent local curvature at critical points of a surface. However, calculating entire Hessians for each form is not a trivial task. As stated above, post-processing by calculating enough elements of a Hessian can be used to detect differences in forms. Hessian solutions could be used as input in a PCA to devise a morphospace in addition to or instead of using Jacobian solutions. Alternatively, solutions to partial Hessians along with solutions to Jacobians can be used as the data to represent models in morphospace analysis.

Whether creating a new set of parametric 3D equations for a model or using solutions to Hessians (or partial Hessians), almost identical forms will plot very close to each other in morphospace. Differences in surface morphology for forms constructed with nearly identical parametric 3D equations may require a post-processing step. Since parametric 3D equations are not invariant, the behavior of the equations from form to form in a more comprehensive way has yet to be tested. Jacobians can be characterized generally as transformation matrices that are used in the linearization of non-linear systems such as the models we constructed using parametric 3D equations. Further testing is necessary and is beyond the purposes and scope of this current study. However, such testing may be necessary for additional analyses such as measuring disparity.

In the future it will be important to explore further how different geometries and surface features are summarized by the Jacobian. In addition, more analysis is needed on the mathematical properties of parametric 3D equations and Jacobians, including testing with regard to boundary conditions, evaluating homogeneous equations at degree *n*, linearization of a non-linear system, evaluating critical points and stability of the Jacobian. These would each be separate studies and are beyond the scope of this paper.

### 4.2. The Utility of Surface Morphospaces

We have demonstrated that parametric 3D models can be meaningfully ordinated using Jacobians, and forms with similar overall surface geometry in terms of tangent lines and planes plot close together in morphospace. The approach presented should be applicable to a broad range of questions where documenting and analyzing scale-invariant patterns of surface form is important. This could include questions related to morphological disparity, functional morphology, ontogeny, and morphological evolution more broadly. In principle, these questions could be explored at a range of taxonomic levels. As our simulations of turritellid ontogeny demonstrate (Fig. 5), even patterns of change at the level of individuals can be studied. The analysis of surfaces potentially provides a richer, more complete assessment of morphology because more of the phenotype may be incorporated when compared to methods based on curves, points, or other one or two dimensional representations of form.

The ability to include disparate forms in a common morphospace is of particular interest and should present new directions for research that have not been pursued before due to methodological limitations [54]. Many existing techniques require some degree of uniformity or similarity in form before analysis can proceed. Applying log spiral models, for example, requires that the phenotypes display spiral geometry; geometric morphometric methods require a common framework of homologous points that precludes the analysis of very different phenotypes. Our method, however, simply requires that the objects have bounded 3D surfaces that are amenable to modeling using parametric 3D equations.

The method shows promise for the study of patterns of ontogeny, but even here it is important to recognize what is being captured in the morphospace. The normally coiled turritellid ontogenetic trajectory (Fig. 5), for example, shows substantial changes as growth proceeds. This may seem counterintuitive as the growth is isometric, and there is no change in the underlying spiral geometry. However, from the perspective of change in surface form this pattern makes perfect sense as each growth stage includes the addition of an entire whorl of *new* shell surface. Compared to a typical bivalve, which does not add whorls during growth, a snail will display much more change in surface geometry during ontogeny. As an aside, it is worth noting that ontogenetic patterns could have been portrayed with each whorl (or growth increment) plotted separately rather than plotting the cumulative morphology. As patterns are scale-invariant, all increments that differ in size only (i.e., isometry) would plot at a single point in space, both for bivalves and gastropods.

While many biologists may be accustomed to studying surfaces or surface features, few have experience describing entire surfaces mathematically and intuiting what surface differences mean. The applicability of our method to a particular question will require a careful consideration of the appropriateness of surface data and the significance of differences in surface geometry (Fig. 6). When analyzing morphological patterns at higher taxonomic levels (e.g., [55]), for example, it may be important to determine if surface morphology is an effective proxy for higher taxa and that distances in morphospace are correlated with taxonomic/phylogenetic rank.

The richness of information embedded in the elements of the Jacobian means that a surface morphospace is, in some ways, more complicated. We hope that this new method will promote the study of biological surfaces and stimulate thoughtful discussion of the concept and use of a morphospace [56]. We expect that comparing phenotypes in terms of their overall surface form in a mathematically rigorous framework will enrich the study of morphology in general, and as the method is applied, new ways in which to treat the data acquired will emerge.

## Conclusions

Parametric 3D equations can be used to accurately model certain biological surfaces. As shown in Figure 1, our approach can be applied to a variety of basic geometric forms and used to model any bounded surface. Although we use mollusks and echinoids as examples, the same approach can be applied to a wide diversity of organismal forms. Our results clearly demonstrate the utility of the Jacobian in describing and analyzing surface morphology. The ability to summarize a broad range of surfaces with a six-element matrix without any loss of resolution or need for preprocessing is a potential advantage over other methods. However, we have also identified some potential limitations. As with any study, it is important to ensure that the method is appropriate for the question being addressed. The flexibility and mathematical rigor of our approach makes it usable alone or in conjunction with other 3D surface analytical methods to address a broad range of questions. Models constructed could be exported as point clouds and analyzed using methods such as eigensurface [1]–[2], spherical harmonics [3] wavelet analysis [4], or geometric morphometrics [5]. The models should permit the analysis of theoretical and empirical data in a common framework using any one of these 3D techniques.

The ability to quantify entire surfaces and construct multivariate surface spaces should generate new questions regarding morphological evolution. Patterns of phenotypic variation, changes in developmental trajectories, simulations of morphospace occupation, including unrealized forms or hypothetical patterns of ontogeny and evolution, can all be studied in the context of surface morphology. Although we emphasize whole surfaces, homologous or otherwise comparable parts can be treated as bounded substructures. The appearance of new parts on the surface does not preclude analysis, and in a sense, our method can be viewed as “homology-free [57].” Experiments involving the addition or removal of evolutionary novelties could be conducted to determine influences on morphological patterns. Our approach offers another avenue to study morphological variation, and more broadly, the potential to study evolutionary and non-evolutionary patterns and processes in new and fruitful ways.
