# Correction: A Generalized Approach to the Modeling and Analysis of 3D Surface Morphology in Organisms

**DOI:** 10.1371/annotation/fc59dde0-057f-4a59-a5ab-9dcc3b949828

**Published:** 2014-01-02

**Authors:** Janice L. Pappas, Daniel J. Miller

There was an error in the legend for Figure 5. The symbols representing modeled clams, modeled scallops, spirals, and isometric turritellid series were incorrect. Please view the correct Figure 5 legend here: http://www.plosone.org/corrections/pone.0077551.001.cn.tif

